# Public health impacts of air pollution from the spatiotemporal heterogeneity perspective: 31 provinces and municipalities in China from 2013 to 2020

**DOI:** 10.3389/fpubh.2024.1422505

**Published:** 2024-08-02

**Authors:** Yizhong Ye, Qunshan Tao, Hua Wei

**Affiliations:** ^1^School of Hospital Economics and Management, Anhui University of Chinese Medicine, Hefei, China; ^2^Key Laboratory of Data Science and Innovative Development of Chinese Medicine in Anhui Province Philosophy and Social, Hefei, China

**Keywords:** public health, air pollution, geographically and temporally weighted regression model, influencing factors, spatiotemporal heterogeneity

## Abstract

Air pollution has long been a significant environmental health issue. Previous studies have employed diverse methodologies to investigate the impacts of air pollution on public health, yet few have thoroughly examined its spatiotemporal heterogeneity. Based on this, this study investigated the spatiotemporal heterogeneity of the impacts of air pollution on public health in 31 provinces in China from 2013 to 2020 based on the theoretical framework of multifactorial health decision-making and combined with the spatial durbin model and the geographically and temporally weighted regression model. The findings indicate that: (1) Air pollution and public health as measured by the incidence of respiratory diseases (IRD) in China exhibit significant spatial positive correlation and local spatial aggregation. (2) Air pollution demonstrates noteworthy spatial spillover effects. After controlling for economic development and living environment factors, including disposable income, population density, and urbanization rate, the direct and indirect spatial impacts of air pollution on IRD are measured at 3.552 and 2.848, correspondingly. (3) China’s IRD is primarily influenced by various factors such as air pollution, economic development, living conditions, and healthcare, and the degree of its influence demonstrates an uneven spatiotemporal distribution trend. The findings of this study hold considerable practical significance for mitigating air pollution and safeguarding public health.

## 1 Introduction

Human health has long been considered paramount for personal development and social progress (1). Globally, air pollution is acknowledged as the fourth leading threat to human health, resulting in inevitable health repercussions and substantial economic burdens (2). Data indicate that 4.47 and 5.05% of daily hospital emergency room visits in China are attributable to PM_1_ (particles with a diameter less than 1 micron) and PM2.5 pollution, respectively (3). Additionally, estimates from the Global Burden of Disease Study suggest that air pollution caused 1.85 million deaths in China, with 1.42 million attributed to particulate matter (PM) pollution (4). Clearly, air pollution has emerged as a significant factor jeopardizing public health in China. Therefore, it is imperative at this stage to conduct specific and accurate empirical studies on the relationship between air pollution and public health to mitigate its detrimental effects.

Researchers in the fields of epidemiology and environmental toxicology have conclusively established that air pollution not only poses a substantial threat to public physical health but also detrimentally impacts mental well-being (5, 6). Specifically, air pollution significantly influences the onset of various diseases, including cardiovascular diseases, cancer, pneumonia, and neurocognitive impairments (7–12). However, these studies have predominantly focused on the individual-level pathogenesis of air pollution on public health, neglecting the influence of other crucial determinants, such as economic development, living environment, and healthcare (13). Additionally, air pollution demonstrates significant negative externalities and regional correlations. Thus, it is imperative to adequately consider the spatial relationship between a region and its neighbors when studying the impacts of air pollution on public health outcomes (14–17). Recently, some scholars have highlighted a strong spatial correlation between air pollution and public health outcomes in China (18). However, further empirical evidence is required to substantiate the spatial impacts of air pollution on public health outcomes in China. Furthermore, researchers in socio-economic and other fields have conducted numerous studies on the relationship between air pollution and public health (19–24). Notably, these studies are commonly analyzed using empirical methods such as ordinary least squares, poisson regression, and generalized linear regression (25–28). While these methods can assess the impacts of air pollution on public health, they tend to rely on the assumption of fixed parameters. The estimation of model parameters is typically confined to either temporal or spatial dimensions individually. This limitation prevents the simultaneous consideration of parameter heterogeneity in both temporal and spatial dimensions, resulting in inaccurate estimates (29). To summarize, after fully considering China’s current reality and existing research gaps, the research motivation of this study is mainly reflected in the following three research questions: (1) How do factors such as air pollution, economic development, and the living environment influence public health in China? (2) What are the spatial impacts of air pollution on public health in China over a long time series at the interprovincial scale? (3) Are there spatiotemporal heterogeneities in the impacts of air pollution on public health from the perspective of regional differences in China?

Based on these considerations, the objectives of this study are multifaceted. First, on the basis of the theoretical framework of multifactorial health decision-making, this study comprehensively and systematically examines the role and influence of air pollution, economic development, and other factors on public health. Second, the spatial impacts of air pollution and related control variables on public health are examined thoroughly by constructing a spatial econometric model. Finally, geographically and temporally weighted regression (GTWR) models were employed to further analyze the spatiotemporal heterogeneity of the effects of air pollution and related control variables on public health. In summary, the potential contributions of this study include the following two main aspects: First, this study reexamines environmental health issues from a socioeconomic perspective and comprehensively analyzes the spatial impacts of air pollution and related control variables on public health, thus enriching the study of air pollution and public health. Secondly, considering the spatiotemporal heterogeneity of the variables used, this study constructs the GTWR model based on spatiotemporal relationships to study the spatiotemporal evolution characteristics of the influencing factors, especially in analyzing the spatiotemporal heterogeneity of interregional public health, which not only illustrates the reality of imbalances of regional economic and resource distributions in China, but also provides a useful supplement to previous related studies.

The remainder of the paper is structured as follows. Firstly, the paper reviews the pertinent literature. Subsequently, it describes the study area, research methodology, variable selection, and data sources. Following this, the study results, including the primary findings, are presented and discussed. Finally, the main conclusions and policy recommendations of the paper are summarized.

## 2 Literature review

### 2.1 The impact of air pollution on public health

Researchers have extensively focused on the impact of air pollution on public health. Scholars in medicine and public health have conducted numerous studies on this relationship. de Prado Bert et al. (30) conducted an epidemiological study confirming that air pollution adversely affects cognitive behavior and psychomotor activity in children due to exposure to air pollution. Based on Lee et al. (31) air pollution significantly exacerbates respiratory symptoms such as asthma and chronic obstructive pulmonary disease. Fu et al. (32) performed a comprehensive exploration of the effects of air pollutants such as particulate matter, ozone, and sulfur oxides on the nervous system, demonstrating that these pollutants can directly or indirectly damage it. Liang et al. (33) used a meta-analysis showing a significant increase in the risk of gestational diabetes due to air pollution. Shi et al. (34) demonstrated that chronic exposure to PM_2.5_ is significantly associated with an increased incidence of dementia in a cohort study. Requia et al. (35) investigated the relationship between daily PM_2.5_, NO_2_, and O_3_ levels and the number of hospitalizations for circulatory and respiratory diseases, showing a significant association between air pollution and hospital admissions for cardiopulmonary diseases. Demoury et al. (36) assessed the association between natural mortality and short-term exposure to air pollutants (PM_2.5_, PM_10_, NO_2_, and O_3_), finding that increased air pollution led to an increase in overall natural mortality. In China, Zhenyu et al. (37) discovered that fine particulate matter (PM_2.5_) significantly increased the risk of lung cancer based on incidence rates and annual concentrations of PM_2.5_. Liu et al. (38) estimated the association between air pollution and asthma mortality, highlighting that PM_1_ pollution had a more significant impact on asthma mortality compared to PM_2.5_ and PM_10_. Zhang et al. (39) evaluated the effect of air pollutant exposure on hospital admissions for congenital heart disease, concluding that exposure during pregnancy significantly increased the risk of congenital heart disease in perinatal infants. Li et al. (40) established a significant positive correlation between the increased risk of stroke hospitalization and short-term elevations of air pollutants such as SO_2_, NO_2_, and PM_10_. In summary, time-series or case-crossover studies detect associations between daily mortality (or morbidity) and changes in air pollution to capture its acute impacts, while cohort and cross-sectional studies reveal the increased risk of health hazards from long-term exposure.

### 2.2 The spatiotemporal impact of air pollution on public health

Notably, as the effects of air pollution frequently extend to adjacent regions, suggesting that air pollution leads to both direct and indirect geographical spillover effects (41). Mohammadi et al. (42) conducted a spatial analysis to explore the relationship between air pollution and mortality, revealing a positive spatial effect of pollutants like O_3_ and NO_2_ on mortality. Kim et al. (43) examined the bivariate correlation between air pollutants and the prevalence of allergic diseases in controlled environments, demonstrating the presence of significant spatial clustering and the need to consider the instability of local relationships when studying the effects of air pollution on disease. Aturinde et al. (44) performed a nationwide study investigating the spatial associations between various air pollutants and the prevalence of cardiovascular disease (CVD), highlighting significant spatial variations in the relationship between ambient air pollution and CVD hospital admissions across Sweden. Yim et al. (45) investigated the impact of PM_10_ particulate matter on the risk of pneumonia, revealing a significant spatial correlation between air pollution and the occurrence of pneumonia. Sarrias and Molina-Varas (46) quantified the spillover effect of PM_2.5_ pollution on emergency room visits for respiratory illnesses, demonstrating a significant spatial effect influence on the increase in overall visits. In China, Cao et al. (47) investigated the correlation between air quality and mortality due to respiratory diseases, showing a significant spatial correlation between PM_2.5_ pollution and respiratory disease mortality across all provinces in China. Zhang et al. (48) examined the interactive impacts of air pollution on public health. The study findings revealed that the escalation of air pollution significantly threatened the health of residents in both local and neighboring areas. Qin et al. (49) further investigated the spatial impacts of urban air pollution on public health, indicating a significant spillover effect of air pollution in urban areas. Additionally, other researchers have studied the diverse impacts of air pollution on public health, particularly in elucidating the spatial variability of influencing factors (50). Specifically, Cardoso et al. (51) assessed the spatial relationship between PM_10_ emissions and lung cancer mortality in the Portuguese region using a geographically weighted regression model. They confirmed that the effect of PM_10_ emissions on lung cancer mortality was higher in the northwestern part of the continent, while it was relatively small in the southern part. Chen et al. (52) explored the spatiotemporal relationship between air pollution and influenza using spatiotemporal weighted regression model. They found that the coefficients of air pollution on the intensity of influenza epidemics were opposite in the eastern and western parts of Fuzhou City in China. Yan et al. (53) assessed the potential spatial relationship between PM_2.5_ pollution and health inequality in China using the multi-scale geographically weighted regression model. They showed that the health inequality caused by PM_2.5_ pollution exhibited a spatial distribution trend of decreasing from east to west. Boakye et al. (54) conducted the multi-scale geographically weighted wegression model analysis of the social and spatial factors of cancer and non-cancer hazards due to air pollution. Their analysis demonstrated that there are spatial differences in exposure to air pollution-induced cancer and non-cancer risk outcomes for various racial and ethnic groups in the United States. Wang and Wang (55) analyzed the spatial variability of factors influencing PM_2.5_ pollution in the Yangtze River Delta region of China using a geographically weighted regression model. The study results indicated spatial heterogeneity in the influence of factors such as population density and the proportion of industries on PM_2.5_ pollution. Li and Managi (56) examined the relationship between subjective well-being and three air pollutants (SO_2_, NO_x_, and PM_2.5_), as well as their spatial variability in Japan using the random-effects-based geographically weighted regression model. They found that these air pollutants were negatively associated with human well-being, and that their negative impacts varied spatially.

Generally, the research methods discussed above can be utilized to assess the impacts of air pollution and related factors on public health. However, they often overlook the spatiotemporal heterogeneity of influencing factors, highlighting a necessity to enhance the precision of these methodologies. Specifically, the fundamental assumption of global regression models such as spatial lag model (SLM), spatial error model (SEM), spatial durbin model (SDM), and ordinary least squares (OLS) is that the relationship between the independent and dependent variables remains spatially consistent and does not vary across different spatial locations (57). This class of models offers a global perspective applicable to all spatial entities. However, significant spatial heterogeneity in air pollution, public health, and related influences may exist in the real world. Therefore, global regression models may yield inaccurate estimates (58). In contrast, geographically weighted regression (GWR) model serves as a localized regression method for exploring the spatial heterogeneity of air pollution, public health, and related influences (59). Specifically, the GWR model offers a novel approach to assessing spatial heterogeneity among spatial entities by computing multiple regression statistics for each entity (60). However, the GWR model inadequately addresses the influence of time series on public health, air pollution, and related factors. One approach to surmounting these challenges is the geographically and temporally weighted regression (GTWR) model proposed by Huang et al. (61). In contrast to the GWR model, this approach enhances estimation accuracy through the incorporation of temporal and non-stationary spatial weighting matrices (62). Based on this, Zhang et al. (63) examined the spatial and temporal correlation between chronic disease co-morbidities and the air pollutant PM_2.5_ among middle-aged and older adults in China from 2011 to 2018 using the GTWR model. Their empirical findings demonstrated the GTWR model superiority over traditional correlation algorithms. Furthermore, PM_2.5_ emerges as the primary risk factor for chronic disease co-morbidity prevalence among middle-aged and older adults. Its impact spans from the southeast coast to the inland regions, though diminishing from the coast towards inland areas. Wang et al. (64) employed the GTWR model to quantitatively assess the relationship between acute myocardial infarction (AMI) mortality and influential factors in China, from 2014 to 2016. Their study revealed that environmental factors, including air pollution, exerted a significant negative impact on AMI mortality, with variations observed across different locations and over time. Yu et al. (65) investigated the temporal and spatial heterogeneity of the relationship between provincial tuberculosis (TB) incidence rates and environmental and economic factors in China from 2004 to 2021 using the GTWR model. Their findings demonstrated that the GTWR model provided a better fit than both ordinary least squares (OLS) and geographically weighted regression (GWR) models. Moreover, TB incidence in China is predominantly influenced by macroeconomic and environmental factors, with varying degrees of impact observed across different times and regions. Overall, the feasibility of employing the GTWR model to evaluate the public health impacts of air pollution and related factors remains a challenge.

### 2.3 The impact of socio-economic and other factors on public health

In fact, the effects of air pollution on public health are influenced by a confluence of factors including economic development, healthcare, and living conditions (66). Consequently, scholars in socio-economic and related disciplines have extensively investigated the repercussions of air pollution on public health. Fischer and Heutel (67) substantiated the presence of a mechanism linking air pollution, economic fluctuations, and mortality by comparing air pollutant indices, unemployment rates, and economic shifts. Greenstone and Hanna (68) examined data regarding air pollution and health in developing countries, revealing a greater impact of air pollution on population health in certain developing nations in contrast to developed ones. Natalie et al. (69) assessed the influence of air pollution on public health using self-reported health data from German residents, showing a notable impact on residents’ self-reported health, particularly exacerbated amidst heightened socio-economic insecurity, unemployment, and residence in deprived areas. Deryugina et al. (70) further demonstrated that the impact of air pollution on mortality in the United States is influenced by healthcare utilization and healthcare costs. In China, Zhang et al. (71) assessed the correlation between outdoor air pollution and older adults’ cognitive function, discovering that the detrimental effects of air pollution to older adults’ cognitive function is influenced by seasonal variation, age, and education. Azimi et al. (72) shared a similar perspective, contending that regional disparities in economic, social, environmental, and healthcare factors result in notable geographical variations in the public health consequences of air pollution across different regions. Wu et al. (73) examined the relationship between household income growth and health risks related to air pollution, demonstrating that increased household income notably mitigates health risks from air pollution, particularly for gaseous pollutants (SO_2_ and NO_x_). Geng et al. (74) meanwhile, quantified the influence of various factors on PM 2.5-related fatalities in China, affirming that alterations in energy and climate policies, as well as economic structure, notably impact the number of deaths linked to PM 2.5. Pang et al. (75) investigated the influence of green space on the incidence of lung cancer associated with air pollution, revealing that areas with higher green space exhibit attenuated adverse effects of air pollution on lung cancer incidence.

### 2.4 Literature summary

The existing literature has thoroughly investigated the impacts of air pollution on public health, establishing a robust foundation for this study. Nonetheless, several pertinent studies in epidemiology and environmental toxicology have been conducted using non-randomized samples of individuals or specific regions. Simultaneously, these studies typically concentrate solely on the pathomechanisms of air pollution’s effects on public health, neglecting other influential factors like economic development and population density (76). Secondly, the existing literature often assumes independence among all public health domains, disregarding the spatial correlation of air pollution. This oversight results in estimation errors regarding the impact of air pollution on public health (77). Finally, in investigating the spatial impacts of air pollution on public health, most prior studies have relied on spatial panel modeling for empirical analysis, overlooking both spatial and temporal variations in influencing factors (78). Although some scholars in China have recently employed the GWR model to examine spatial variations in influencing factors, their failure to explore the temporal evolution of these factors has hindered a comprehensive explanation of their impacts.

Consequently, to address the limitations of prior studies and mitigate potential estimation bias and inconsistency, we enhance several aspects as follows. Firstly, based on the theoretical framework of multifactorial health decision-making, we constructed a spatial econometric model for influencing public health. Through this model, we can comprehensively assess the impacts of air pollution, economic development, and other factors on public health, and delve into the spatial correlation between air pollution and public health, thereby effectively mitigating estimation bias arising from endogeneity and overlooking spatial effects. Secondly, on a long-term interprovincial scale, this study provides a more rigorous interpretation of both direct and indirect spatial effects of air pollution, thereby enhancing the analysis of spatial impacts on public health and ensuring the scientific validity of the conclusions. Finally, this paper introduces the GTWR model to compare and analyze temporal changes in influencing factors across an eight-year time series, carefully considering local spatial heterogeneity. This approach aims to provide a more detailed and comprehensive understanding of the relationship between air pollution and public health across temporal and spatial scales.

## 3 Theoretical framework

In fact, scholars in socio-economics and related fields have demonstrated that many factors affect public health, especially the economy, environment, education, and healthcare (79). Therefore, we used Neidell’s theoretical framework of multifactorial health decision-making as a basis for describing the mechanisms by which air pollution, social, economic, and educational factors affect public health (80). Fundamentally, the framework asserts that health is not merely the result of biological factors or self-selection but is strongly influenced by a wide range of factors, including economic, social, environmental, and educational factors, as well as air pollution. These influences dynamically interact, resulting in a complex network that affects public health (81).

Specifically, economic factors are important determinants in the framework, including income levels, employment status, socio-economic status, and access to resources. Economic stability allows individuals to afford quality healthcare, nutritious food, and safe housing, all of which are necessary to maintain good health (82). In contrast, economic hardship can lead to poor living conditions and limited access to healthcare, each of which negatively impacts public health (83). For example, people of lower socio-economic status often face barriers to accessing healthcare services and promoting personal health, leading to delays in treatment, ineffective management of chronic diseases, and deterioration of health status (84).

Social factors play an essential role in shaping health outcomes, which including social support networks, community participation, cultural practices and social norms (85). On the one hand, strong social support networks can provide emotional support, reduce stress and encourage healthy behaviors in the general public (86). Additionally, the enhancement of public community participation fosters an individual’s sense of social belonging and purpose, which in turn promotes his or her mental and emotional health (87). On the other hand, cultural practices and social norms can promote or hinder the emergence of healthy behaviors in citizens (88). For instance, social norms that ignore mental health problems may exacerbate the emergence of mental health problems by preventing individuals from seeking help (89).

Environmental factors, like the quality of air and water and access to green spaces, which are crucial factors affecting public health. Poor environmental conditions such as severe air pollution may contribute to the development of respiratory diseases, cardiovascular problems and other health problems (90). On the contrary, having access to clean air, clean drinking water and green space for physical activity promotes public health and social well-being (91). In addition, scholars in the fields of epidemiology and environmental toxicology have noted that exposure to air pollutants can produce a range of health problems, from asthma and allergies to more serious diseases such as lung cancer and heart disease (92). Thus, the framework specifically emphasizes air pollution as an important environmental factor affecting public health.

Educational factors mainly include educational attainment, health literacy and the opportunity to receive health education. In general, higher levels of education are usually positively associated with better health outcomes. The reason for this is that higher levels of education usually equip individuals with relevant health knowledge and skills, which in turn promotes the development of healthy behaviors (93). Simultaneously, health literacy is also particularly important. This is because it enables individuals to attend to their own health information and engage in preventive health behaviors (94). Furthermore, increased access to health education can empower individuals to take control of their health, leading to better health outcomes (95).

The framework underscores the pivotal role of healthcare services in influencing public health. The healthcare system delivers essential services, including prevention, diagnosis, and treatment of diseases. Thus, access to quality and efficient healthcare is crucial for individuals to manage their health conditions and prevent complications (96). Simultaneously, an effective healthcare system not only treats diseases but also supports public health through integrated care that addresses physical, mental, and social needs (97). Additionally, the interaction between healthcare services and diseases is a central element of the framework. Specifically, disease conditions affect the healthcare needs of the public, while the availability and quality of healthcare services determine how efficiently these needs are met (98). Thus, the framework posits that mitigating the negative impact of diseases on an individual’s health functioning and health status can be achieved by improving the accessibility and quality of healthcare (99).

**Figure 1:**
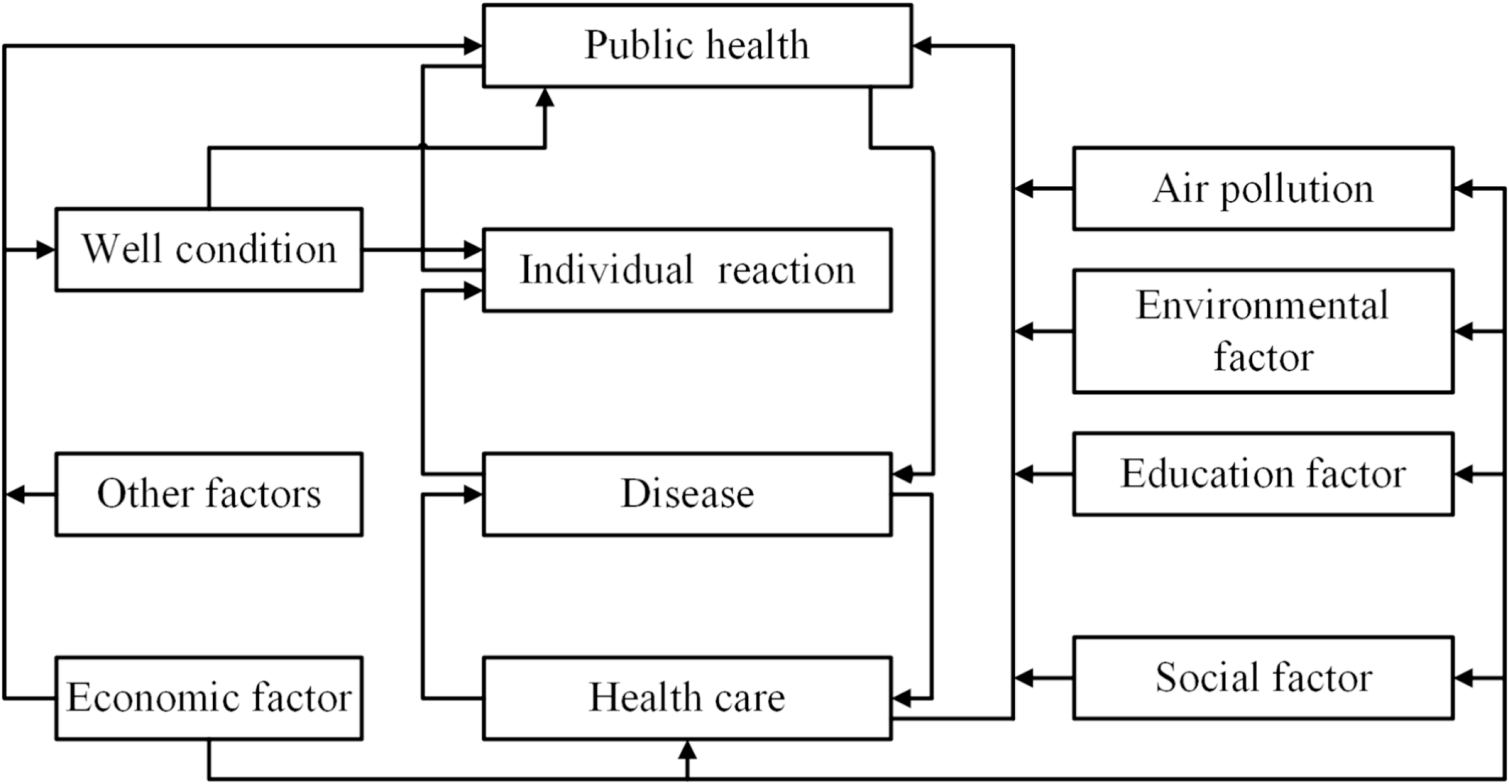
The theoretical framework of multifactorial health decision-making (80).

Furthermore, the framework further emphasizes the interconnectedness of various influences on health. This means that economic, social, environmental, and educational factors affecting public health do not exist in isolation but rather interact to influence health outcomes (100). For example, economic stability can increase citizen access to education, which in turn improves their health literacy and promotes the occurrence of healthy behaviors (101). Social support networks can promote physical and mental health by providing emotional support, alleviating the negative effects of economic hardship and environmental stress (102). Improving environmental quality and reducing air pollution can create a fairer and healthier environment, particularly for vulnerable groups affected by poor environmental conditions (103). From above, the multifactorial health decision-making theoretical framework is shown in Figure 1.

## 4 Materials and methods

### 4.1 Variable selection

According to the theoretical framework of multifactorial health decision-making, public health is influenced by healthcare, environmental factors, and economic development level. Based on this, the selected indicators are as follows.

#### 4.1.1 Public health variables

Health is a concept with complex intrinsic mechanisms, which makes it challenging to quantify health. Previous research has mainly selected indicators such as population mortality rate, mean life expectancy, and disability-adjusted life expectancy (DALY) to evaluate public health (104–106). However, it is not appropriate to use population mortality rate to assess the impact of air pollution on public health. This is because the health effects of air pollution are relatively slow and the causes of population mortality are usually complex. In addition, the absence of provincial-level data on mean life expectancy and DALY in China makes it challenging to perform effective spatial econometric analyzes. Thus, we used a surrogate indicator, the incidence of respiratory diseases (IRD). Previous studies have shown that the indicators most associated with air pollution are respiratory ailments (107). Heightened air pollution exacerbates the likelihood of respiratory and circulatory ailments within the populace (108, 109). Consequently, we chose IRD to assess the level of public health so as to accurately identify the causal relationship between air pollution and public health.

#### 4.1.2 Air pollution variables

Scholars have employed various evaluation indicators, including PM_2.5_ concentration, soot emissions, and sulfur dioxide emissions, to assess air pollution (110–112). However, a single pollutant index may inadequately capture the comprehensive nature of air pollution, thereby neglecting the cumulative impact of multiple pollutants on public health. Consequently, the air pollution index (API) was employed in this study to evaluate air pollution in China (113). The API is a non-linear dimensionless index derived from a comprehensive calculation of the concentrations of various air pollutants (including PM_2.5_, Soot, SO_2_, and NO_x_) by the entropy method. A higher API value indicates greater air pollution severity and poses increased risks to public health.

#### 4.1.3 Control variables

Previous research has shown that various factors such as economic development, living environment, and healthcare also influence public health (114–118). Thus, we selected specific indicators as control variables: economic development, represented by resident disposable income (RDI); aspects of the living environment, including population density (PD), greening coverage (GRE), and urbanization rate (URB); and healthcare, denoted by the number of occupational physicians per 1,000 people (OCP) and household medical expenditure (HME).

#### 4.1.4 Data sources

**Figure 2:**
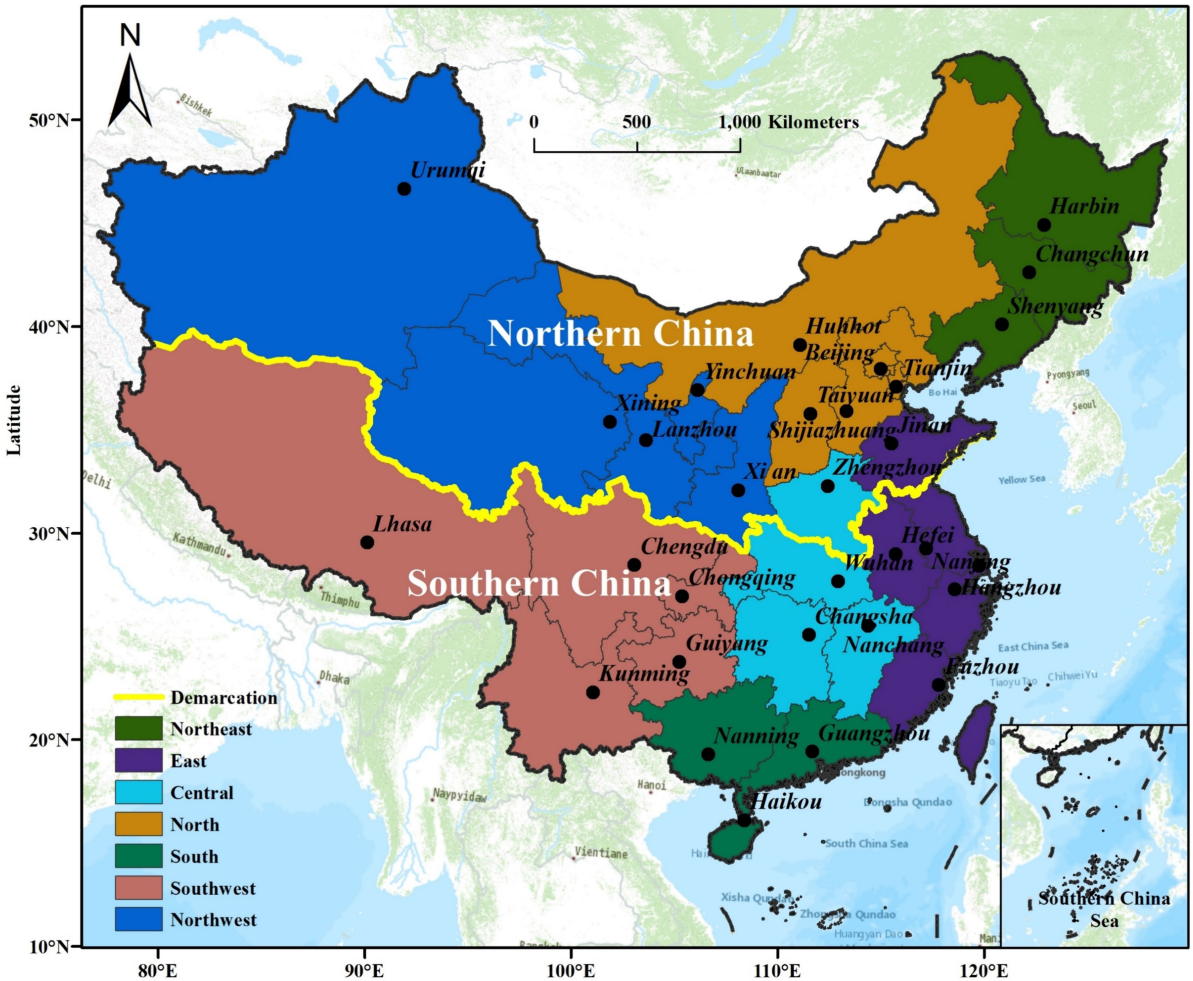
Research areas and the division of northeast, east, central, north, south, southwest and northwest regions of China.

The study’s data encompasses 31 provinces and municipalities in China (including autonomous regions and municipalities directly under the central government), excluding Hong Kong, Macao, and Taiwan. These are omitted because the majority of the data is missing. Meanwhile, according to China’s administrative regions, the study areas encompass Northeast, East, Central, North, South, Southwest, and Northwest, as illustrated in Figure 2.

Furthermore, the primary data sources for the study were the China Statistical Yearbook, hyperlink to datasets source,^1^ China Health and Sanitation Statistical Yearbook, hyperlink to datasets source,^2^ China Environmental Statistical Yearbook, and China Ecological and Environmental Condition Bulletin, hyperlink to datasets source.^3^ Meanwhile, natural logarithms were applied to all variables to mitigate the impact of heteroscedasticity on the regression outcomes. Table 1 presents the details of each variable along with their descriptive statistics.

**Table 1:** Outline of the variables.

Index	Specific description	Unit	Mean	Standard deviation	Minimum	Maximum
Public health	Incidence of respiratory diseases (IRD)	Persons per ten thousand persons	5.313	1.029	2.116	7.104
Air pollution	Air pollution index (API)	–	0.222	0.146	0.003	0.901
Socioeconomic variables	Resident disposable income (RDI)	Yuan	10.05	0.379	9.184	11.19
	Population density (PD)	Person/km^2^	5.332	1.496	0.948	8.275
Life Environment variables	Urbanization rate (URB)	%	4.061	0.217	3.175	4.495
	Greening coverage (GRE)	%	3.579	0.116	2.526	3.85
Healthcare variables	Number of occupational physicians per 1,000 people (OCP)	Persons per thousand persons	0.701	0.258	0.03	1.705
	Household medical expenditure (HME)	Yuan	7.396	0.374	6.281	8.288

### 4.2 Methods

#### 4.2.1 Spatial econometric analysis

Air pollution is often characterized by spatial spillover and dispersion, where air pollutants flow from one province to neighboring provinces, creating public health hazards of varying degrees in these areas (119). Therefore, this study investigates the macro-level spatial effects of air pollution on public health utilizing a spatial econometric model.

##### 4.2.1.1 Spatial autocorrelation

Spatial autocorrelation refers to the phenomenon wherein a specific economic phenomenon or attribute within an economic region exhibits correlation with similar phenomena or attribute values in neighboring economic regions, including both global and local spatial autocorrelation (120). Among these, global spatial autocorrelation is evaluated using Moran’s I, which is calculated as:


(1) $$I=\frac{\sum_{i=1}^{n}\sum_{j=1}^{n}W_{i,j}=\left(x_{i}-\bar{x}\right)\left(x_{j}-\bar{x}\right)}{S^{2}\sum_{i=1}^{n}\sum_{j=1}^{n}W_{i,j}}$$

In Eq. 1, *I* represents the overall extent of correlation between regions; n represents the number of study regions; $x_{i}$ and $x_{j}$ represent the data points in regions i and j , respectively; $\bar{x}$ represents the mean of the data points; $S^{2}=\frac{1}{n}\sum_{i=1}^{n}{\left(x_{i}-\bar{x}\right)}^{2}$ represents the variance of the sample; and $W_{i,j}$ represents the spatial weighting matrix. Additionally, considering that the effects of air pollution on public health can be experienced across regions, this paper adopts the geographical neighborhood weight matrix to calculate Moran’s I. The assignment criterion of the geographical neighborhood weight matrix is as follows: 1 indicates that regions i and j are neighbors spatially, and 0 indicates that region i and region j are not neighbors spatially. The formula is as follows:


(2) $$W_{i,j}=\{\begin{array}{c} 1,\mathrm{the}\;\mathrm{region}\;i\;\mathrm{is}\;\mathrm{adjacent}\;to\;\mathrm{the}\;\mathrm{region}\;j;\\ \;0,\mathrm{the}\;\mathrm{region}\;i\;\mathrm{is}\;\mathrm{not}\;\mathrm{adjacent}\;to\;\mathrm{the}\;\mathrm{region}\;j. \end{array}\left(i\neq j\right)$$

In addition, the range of Moran’s I is [−1, 1], if the index value approaches 1, it indicates stronger spatial positive correlation; if the index value approaches −1, it indicates stronger spatial negative correlation; if the index value equals 0, it indicates spatially random distribution.

##### 4.2.1.2 Local spatial autocorrelation analysis

Local spatial autocorrelation analysis is pivotal in understanding spatial patterns within datasets. Global spatial autocorrelation analysis solely indicates the presence of spatial correlation within an economic phenomenon or attribute, but lacks the capacity to delineate the spatial agglomeration characteristics. Therefore, Anselin conducted additional analysis to evaluate the significance of spatial clustering using the local spatial autocorrelation measure, Local Moran’s I (121). The formula for Local Moran’s I is presented below.


(3) Moran'sIi=nxi−x¯∑i=1nxi−x¯2∑j=1nWi,jxj−x¯

In Eq. 3, the variables carry the same significance as those in Eq. 2. If Moran'sIi>0 , it indicates that the province shares similar attributes with its neighboring provinces; if Moran'sIi≤0 , it indicates the absence of similar attributes with its neighboring provinces. Furthermore, we employ the LISA agglomeration map to depict spatial relationships between regional units and their neighbors. This allows us to categorize these relationships into four types of spatial correlations: high-high agglomeration (H-H), high-low agglomeration (H-L), low-high agglomeration (L-H), and low-low agglomeration (L-L).

##### 4.2.1.3 Spatial measurement models

According to LESAGE et al. and other researchers, spatial autoregressive (SAR), spatial error model (SEM), and spatial durbin model (SDM) are frequently employed in spatial econometric analysis (122). The SAR model examines spatial spillover effects of explanatory variables, incorporating a spatial lag term; whereas the SEM model investigates spatial dependence due to omitted variables, incorporating a spatial lag term in the error term. The SDM model integrates the benefits of both SAR and SEM by addressing spatial dependence of explanatory variables and accounting for spatial effects caused by random errors (123). Based on this, the expression for the aforementioned model is as follows:

Spatial autoregressive econometric modeling (SAR):


(4) $$LnY_{\mathrm{it}}=\rho \overset{n}{\underset{j=1}{\sum }}W_{ij}\ln Y_{\mathrm{it}}+\overset{f}{\underset{k=1}{\sum }}\alpha_{k}\ln X_{ik,t}+\mu_{\mathrm{it}}+\lambda_{\mathrm{it}}+\varepsilon_{\mathrm{it}}$$

Spatial error economics modeling (SEM):


(5) $$LnY_{\mathrm{it}}=\beta +\overset{f}{\underset{k=1}{\sum }}\alpha_{k}\ln X_{ik,t}+\mu_{\mathrm{it}}+\lambda_{\mathrm{it}}+\phi_{\mathrm{it}}$$

(6) $$\phi_{\mathrm{it}}=\sigma \overset{n}{\underset{j=1}{\sum }}W_{ij}\phi_{jt}+\varepsilon_{\mathrm{it}}$$

Spatial durbin economics modeling (SDM):


(7) $$\begin{array}{l} LnY_{\mathrm{it}}=\beta +\rho \overset{n}{\underset{j=1}{\sum }}W_{ij}LnY_{\mathrm{it}}+\overset{f}{\underset{k=1}{\sum }}\alpha_{k}LnX_{ikt}\\ +\overset{f}{\underset{k=1}{\sum }}\theta_{k}\overset{n}{\underset{j=1}{\sum }}W_{i,j}LnX_{ijk,t}+\mu_{\mathrm{it}}+\lambda_{\mathrm{it}}+\varepsilon_{\mathrm{it}} \end{array}$$

In Eqs 4–7, $Y_{\mathrm{it}}$ represents the observed value of the explanatory variable for each spatial cell $\left(i=1,\ldots ,N\right)$ at time $\left(t=1,\ldots ,T\right)$ , while $X_{\mathrm{it}}$ represents the observed value of the explanatory variable for the same spatial cell $\left(i=1,\ldots ,N\right)$ at time $\left(t=1,\ldots ,T\right)\text{.}$ The symbol β signifies the intercept term. i represents province i , j represents province j ; t signifies the year; ρ denotes the spatial autoregressive coefficient, indicating the spatial correlation among sample observations; σ represents the spatial error coefficient, indicating the influence of residuals from neighboring regions on local residuals; $W_{ij}$ denotes an entry in the geographic neighborhood weight matrix (124); $\mu_{\mathrm{it}}$ signifies the temporal fixed effect of a spatial unit; $\lambda_{\mathrm{it}}$ signifies the individual fixed effect of a spatial unit; $\varepsilon_{\mathrm{it}}$ denotes the random disturbance term. Finally, $\phi_{\mathrm{it}}$ represents the spatially correlated error term.

Furthermore, LeSage et al. (125) has highlighted that the presence of spatial correlation hinders the coefficients of the explanatory variables in the SDM from accurately reflecting the marginal effects. A more accurate interpretation of the model can only be achieved by decomposing the marginal effects into direct, indirect, and total effects. Specifically, the direct effect reflects the impact of the explanatory variables in the region on the local explanatory variables, while the indirect effect signifies the influence of the explanatory variables in neighboring regions on the local explanatory variables. The combination of direct and indirect effects is termed as the total effect (126).

On the basis of the above analysis, we select the SDM to explore the impact of air pollution on public health. The specific formula is as follows:


(8) $$\begin{array}{l} LnH_{\mathrm{it}}=\beta +\rho W_{ij}LnH_{\mathrm{it}}+\alpha_{1}\mathrm{LnAP}I_{\mathrm{it}}+\alpha_{2}LnX_{\mathrm{control}}\\ +\theta_{1}W_{ij}\mathrm{LnAP}I_{\mathrm{it}}+\theta_{2}W_{ij}LnX_{\mathrm{control}}+\mu_{\mathrm{it}}+\lambda_{\mathrm{it}}+\varepsilon_{\mathrm{it}} \end{array}$$

In Eq. 8, $LnH_{\mathrm{it}}$ represents the explained variable denoting public health status in the ith province during period t ; $LnX_{\mathrm{control}}$ serves as a control variable encompassing factors like economic development and healthcare coverage; $\mathrm{LnAP}I_{\mathrm{it}}$ represents the core explanatory variable signifying air pollution levels in the ith province during period t ; and the remaining variables, denoted by $\beta ,\rho ,\alpha_{k},\theta_{k},\mu_{\mathrm{it}},\lambda_{\mathrm{it}},\varepsilon_{\mathrm{it}},\;\mathrm{and}\;W_{ij}$ , carry the same interpretation as in Eq. 7.

#### 4.2.2 Geographically and temporally weighted regression

The Geographically Weighted Regression model is a local regression technique used to assess the spatial non-stationarity of continuous parameter surfaces at a regional scale. Specifically, the GWR model generates unique regression equations for individual sample points, allowing for localized parameter estimation instead of global parameters (127). Consequently, the use of the GWR model can significantly enhance the accuracy of model estimation, which is particularly beneficial for studying China, a country with a complex spatial background (128). However, the GWR model can only be used for cross-sectional data and cannot adequately consider the temporal dynamics of the influencing factors (129). Thus, Huang et al. (61) proposed the Geographically and Temporally Weighted Regression (GTWR) model, integrating temporal effects, to address both temporal and spatial nonstationarities in the data. Based on this, we employed the GTWR model to assess the influence of various factors on IRD across different regions and time periods. Notably, the shadow weights of other sample points on the regression sample points in the GTWR model are determined by constructing a spatiotemporal weight matrix. Thus, the spatio-temporal weight matrix formulated by the GTWR model using temporal and spatial distances is represented as follows.


(9) $$Y_{i}=\alpha_{0}\left(\mu_{i},\theta_{i},t_{i}\right)+\underset{k}{\sum }\alpha_{k}\left(\mu_{i},\theta_{i},t_{i}\right)X_{ik}+\varepsilon_{i}$$

In Eq. 9, $Y_{i}$ represents the explanatory variable, while $\mu_{i}$ and $\theta_{i}$ correspond to the latitude and longitude of the ith observation, respectively. $\left(\mu_{i},\theta_{i},t_{i}\right)$ symbolizes the time series of the ith observation. $\alpha_{0}\left(\mu_{i},\theta_{i},t_{i}\right)$ denotes the regression intercept; $\alpha_{k}\left(\mu_{i},\theta_{i},t_{i}\right)$ represents the regression coefficient associated with the k th explanatory variable at the ith observation; signifies the value of the k th explanatory variable at the ith data point; and $\varepsilon_{i}$ represents the residual error term.

In addition, the main challenge in using the GTWR model is to provide parameter values of $k\left(\mu_{i},\theta_{i},t_{i}\right)$ for variables k and i . To address this challenge, we use the following matrix $k\left(\mu_{i},\theta_{i},t_{i}\right)$ to compute the parameter values of:


(10) $$\alpha^{⌢}\left(\mu_{i},\theta_{i},t_{i}\right)={\left[X^{T}W\left(\mu_{i},\theta_{i},t_{i}\right)X\right]}^{-1}X^{T}W\left(\mu_{i},\theta_{i},t_{i}\right)Y$$

**Figure 3:**
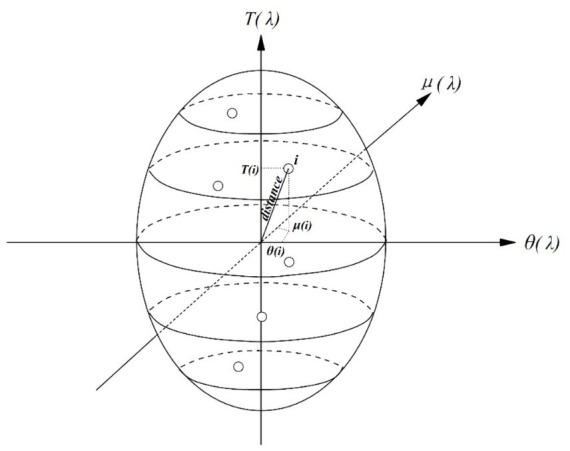
Schematic of the GTWR model spatiotemporal distance.

Eq. 10 involves the diagonal element $\left(\beta_{i1},\beta_{i2},\beta_{i3}\ldots .\beta_{\mathrm{in}}\;\right)$ , which is a function of the spatiotemporal distance $\left(\mu ,\theta ,t\right)$ . Consequently, we calculate the spatiotemporal distances between the regression points and the measured data in this study employing the ellipsoidal coordinate system, which considers variations in locations and times, as illustrated in Figure 3.

Considering the aforementioned information, if we relate the temporal distance, denoted as $\mathrm{distanc}e^{T}$ , to the spatial distance, denoted as $\mathrm{distanc}e^{S}$ , then the spatiotemporal distance, denoted as $\mathrm{distanc}e^{ST}$ , is defined as follows:


(11) $$\mathrm{distanc}e^{ST}=\mathrm{distanc}e^{T}⊗\mathrm{distanc}e^{S}$$

In Eq. 11, ⊗ denotes the disparity between operational symbols. Furthermore, the matrices $\mathrm{distanc}e^{ST}$ and the diagonal elements $W\left(\mu_{i},\theta_{i},t_{i}\right)$ , utilized for the estimation of $\beta_{ij}$ , are expressed in the subsequent equations.


$$\left\{ \begin{array}{c} {\left(\mathrm{distanc}e_{ij}^{S}\right)}^{2}={\left(\mu_{i}-\mu_{j}\right)}^{2}+{\left(\theta_{i}-\theta_{j}\right)}^{2}\\ {\left(\mathrm{distanc}e_{ij}^{T}\right)}^{2}={\left(t_{i}-t_{j}\right)}^{2}\\ {\left(\mathrm{distanc}e_{ij}^{ST}\right)}^{2}=\phi^{S}\left[{\left(\mu_{i}-\mu_{j}\right)}^{2}+{\left(\theta_{i}-\theta_{j}\right)}^{2}\right]+\phi^{T}{\left(t_{i}-t_{j}\right)}^{2} \end{array}\right.$$

(12) $$\begin{array}{c} \beta_{ij}=EXP\left\{-\left(\frac{\phi^{S}\left[{\left(\mu_{i}-\mu_{j}\right)}^{2}+{\left(\theta_{i}-\theta_{j}\right)}^{2}\right]+\phi^{T}{\left(t_{i}-t_{j}\right)}^{2}}{h_{ST}^{2}}\right)\right\}\\ =EXP\left\{-\left(\frac{{\left(\mathrm{distanc}e_{ij}^{S}\right)}^{2}}{h_{S}^{2}}+\frac{{\left(\mathrm{distanc}e_{ij}^{T}\right)}^{2}}{h_{T}^{2}}\right)\right\}\\ ={\beta_{ij}}^{S}\times {\beta_{ij}}^{T\text{.}} \end{array}$$

In Eq. 12, h denotes the bandwidth, a parameter exceeding 0, whereas $h_{S},h_{T}$ , and $h_{ST}$ denote the temporal, spatial, and spatiotemporal bandwidths, respectively. Additionally, $\phi^{S}$ and $\phi^{T}$ represent spatial and temporal distances with scaling factors, respectively. During the computation process, we acquire the time, space, and spatiotemporal bandwidths automatically utilizing cross-validation optimization techniques, and we rely on either the least squares method (Eq. 13) of the R^2 statistic or the modified Akaike information criterion.


(13) $$\mathrm{CVRSS}\left(h\right)=\underset{i}{\sum }{\left(\left(y_{i}-\hat{y_{\neq 1}}\right)h\right)}^{2}$$

In Eq. 13, $\mathrm{CVRSS}\left(h\right)$ denotes the sum of squared errors, while $\hat{y_{i}}\left(h\right)$ signifies the estimated value of $y_{i}$ within the GTWR model.

## 5 Results and discussion

### 5.1 Results of global autocorrelation analysis

**Figure 4:**
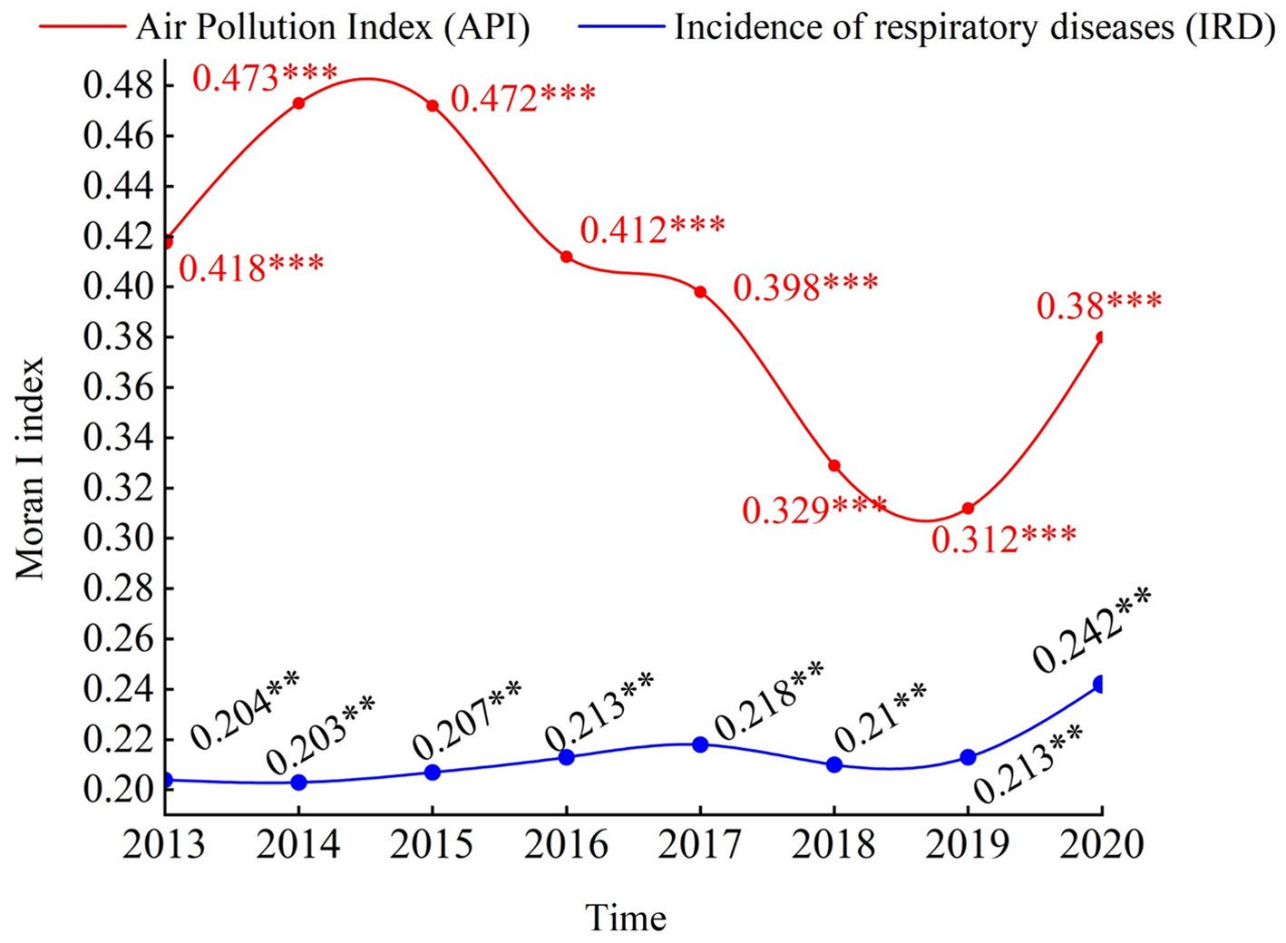
Spatial correlation analysis of air pollution and incidence of respiratory diseases in China, 2013–2020. *, **, and *** distributions indicate 10, 5, and 1% significance levels.

We calculated the global Moran’s I index using Stata17 software to evaluate the spatial correlation between API and IRD, and the results are shown in Figure 4.

As shown in the figure, it illustrate consistently positive Moran’s I values for IRD, all statistically significant at the 5% level. It suggests a significant positive spatial correlation among IRD values, indicating spatial clustering or dependency among provinces with similar public health levels. Specifically, the Moran’s I value for IRD rose from 0.204 in 2013 to 0.242 in 2020. On the other hand, the Moran’s I values for API are consistently positive and pass the significance test at the 1% level, indicating a positive spatial correlation for API. The Moran’s I value for API declined from 0.418 in 2013 to 0.38 in 2020. It is noteworthy that the spatial positive correlation for API is stronger than that for IRD. Thus, when examining the impact of API on IRD, emphasis should be placed on spatial correlations between regions.

### 5.2 Results of local autocorrelation analysis

**Figure 5:**
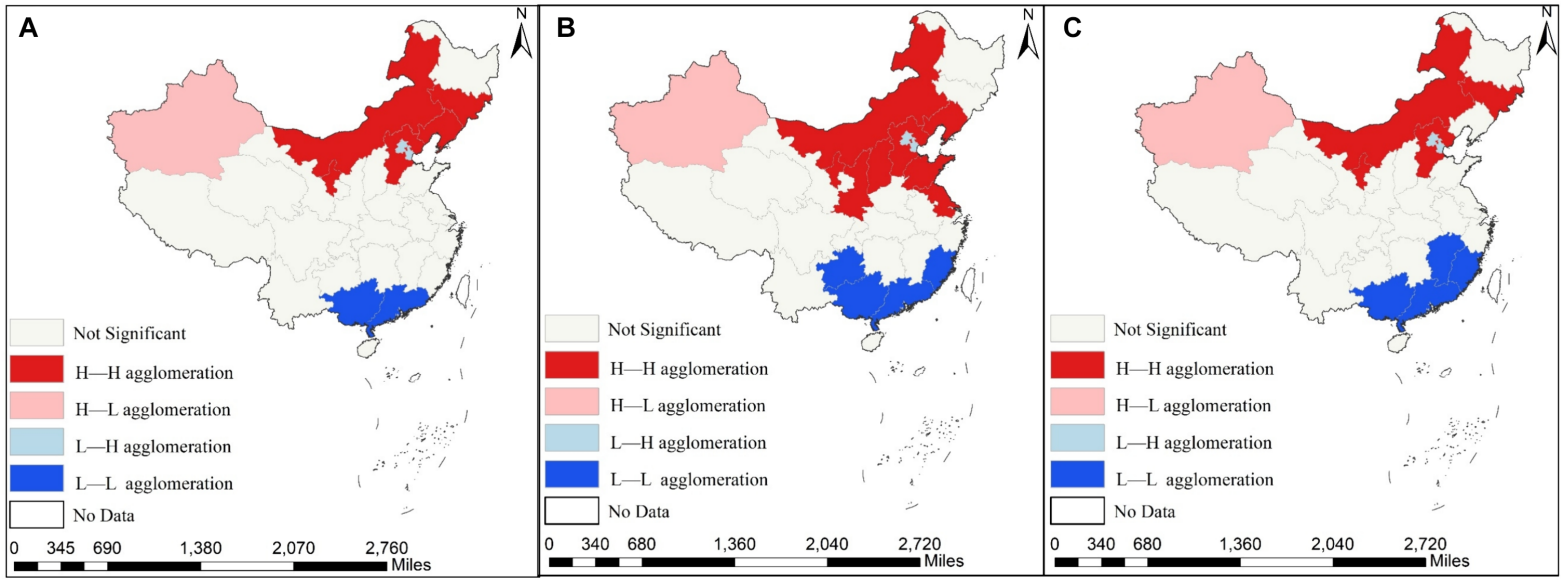
Spatial agglomeration distribution of air pollution index (API) across the 31 provinces of China in 2013, 2016 and 2020. **(A)** Depicts the spatial agglomeration of the API in 2013; **(B)** reflects the spatial agglomeration of the API in 2016; **(C)** indicates the spatial agglomeration of the API in 2020.

**Figure 6:**
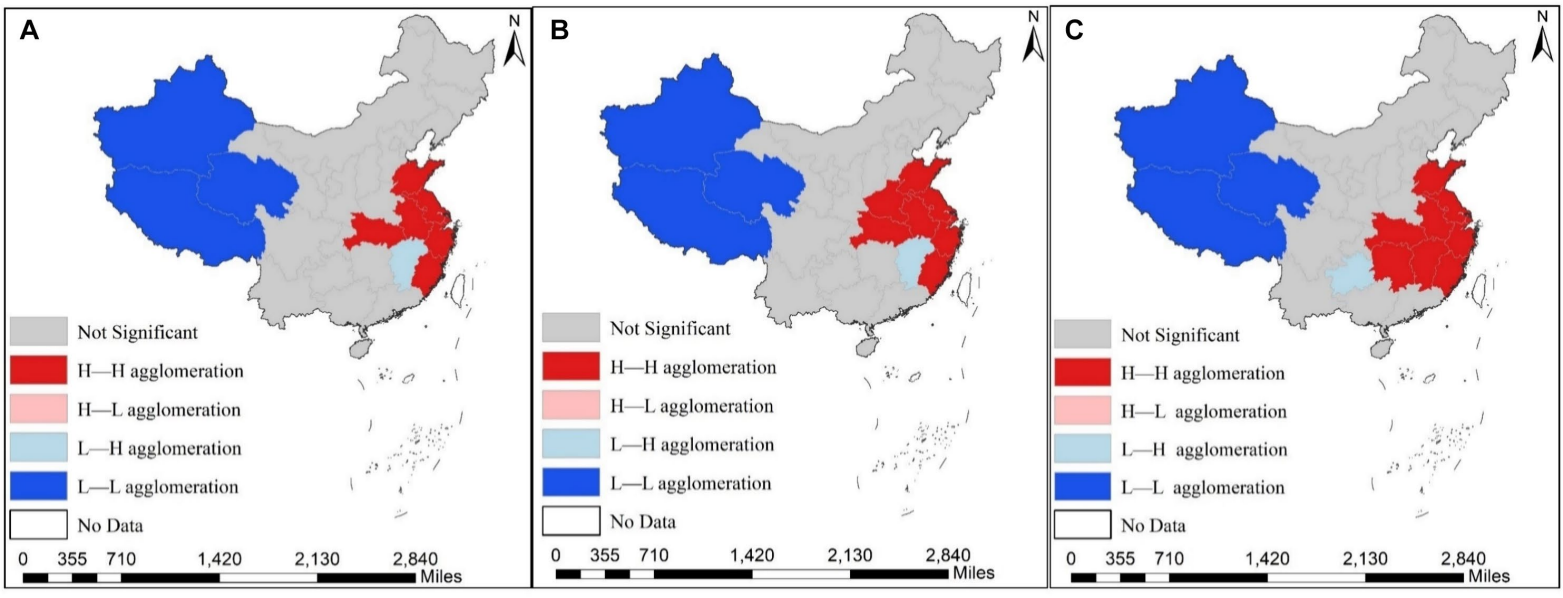
Spatial agglomeration distribution of the incidence of respiratory diseases (IRD) across the 31 provinces of China in 2013, 2016 and 2020. **(A)** Shows the spatial agglomeration of IRD in 2013; **(B)** presents the spatial agglomeration of IRD in 2016; **(C)** displays the spatial agglomeration of IRD diseases in 2020.

Although the Moran’s I index serves as a correlation metric for assessing overall spatial correlation, it does not elucidate the local spatial clustering characteristics between API and IRD. Consequently, LISA clustering maps for 2013, 2016, and 2020 were generated using the global Moran’s I index to depict the local spatial clustering traits of IRD and API. These findings are illustrated in Figures 5, 6.

As can be seen from the figure, the local clustering characteristics of IRD and API during the study period, exhibiting predominantly High-High and Low-Low clustering, which are distributed in adjacent regions. Regarding the API, High-High clustering provinces are mainly found in the central regions of Shanxi and Shaanxi, as well as Xinjiang and Inner Mongolia in the western region, while Low-Low clustering is primarily concentrated in Guangxi, Guangdong, and Fujian provinces. Regarding the IRD, High-High clustering provinces are distributed in Anhui, Zhejiang, and Hunan, as well as Jiangsu and Shanghai in the central and eastern coastal regions. Low-low clustering provinces are mainly concentrated in Tibet, Qinghai, and other western regions. In summary, a clear positive spatial correlation exists between IRD and API. Thus, the utilization of a spatial econometric model for empirical research is warranted.

### 5.3 Estimation and analysis of the spatial durbin model

Before conducting spatial econometric analysis, it is imperative to ascertain the appropriate spatial econometric model through LM test (77, 130). The results of the calculations are presented in Table 2.

**Table 2:** Regression results of the spatial durbin model.

Variables	Model 1 (Temporal-FE)	Model 2 (Spatial-FE)	Model 3 (Spatial–temporal -FE)
LnAPI	3.195***	−0.445***	−0.45***
	(0.375)	(0.0822)	(0.0794)
LnRDI	2.319***	1.274***	1.479***
	(0.309)	(0.225)	(0.244)
LnGRE	−0.561	−0.121**	−0.155***
	(0.397)	(0.0598)	(0.0577)
LnURB	−1.032**	0.328	0.445**
	(0.427)	(0.203)	(0.195)
LnPD	0.565***	−0.331	−0.315
	(0.0490)	(0.219)	(0.222)
LnOCP	−1.448***	0.0348	0.0240
	(0.301)	(0.0595)	(0.0562)
LnHME	−0.169	0.152***	0.111***
	(0.242)	(0.0399)	(0.0384)
W*LnAPI	−2.142***	0.170	0.018
	(0.763)	(0.120)	(0.124)
W*LnRDI	−2.241***	−1.962***	−0.348
	(0.636)	(0.247)	(0.618)
W*LnGRE	−0.713	0.163	−0.065
	(0.868)	(0.127)	(0.129)
W*LnURB	3.748***	0.0396	−0.022
	(0.814)	(0.325)	(0.413)
W*LnPD	0.0375	1.603***	1.438***
	(0.0940)	(0.372)	(0.429)
W*LnOCP	−0.837	−0.0212	0.069
	(0.576)	(0.103)	(0.104)
W*LnHME	−1.173***	0.285***	0.118
	(0.441)	(0.0785)	(0.081)
R^2^	0.616	0.347	0.275
σ^2^	0.219***	0.0022***	0.002***
LM lag	0.352	0.425	0.04
LM error	252.392***	43.809***	30.703***
Robust LM lag	0.03	0.862	0.003
Robust LM error	252.065***	44.247***	30.665***
LR_error	18.26**		
LR_lag	24.35***		
Wald_error	77.13***		
Wald_lag	38.29***		

Standard errors in parentheses. *** *p* < 0.01, ** *p* < 0.05, * *p* < 0.1.

From the table, it is evident that both the LM error and robust LM error tests passed the significance test at the 1% significance level, in comparison with the LM lag and robust LM lag tests, indicating the suitability of the SEM for this study. However, the LM test does not account for the applicability of the SDM. Thus, LR and Wald tests were conducted to assess whether the SDM can be degraded to SAR or SEM. The corresponding statistical *p*-values of both LR and Wald tests, as shown in Table 2, are less than 0.01, significantly rejecting the original hypothesis that the SDM can be degraded to SAR and SEM. Additionally, the Hausman test for the SDM also significantly rejected the random effects hypothesis, leading to the selection of the SDM with fixed effects for analysis. Based on the regression results of different fixed-effects SDM presented in Table 2, Model 1 (Time-Fixed-Effects SDM) demonstrated optimal statistical properties ( $R^{2}=0.616$ ). Therefore, this paper employs the Time-Fixed-Effects SDM as the empirical benchmark.

On the basis of the above analysis, we utilized Stata 17 software to regress the spatial effects of air pollution on public health in 31 Provinces in China from 2013 to 2020. The estimation results are shown in Table 2. As can be seen from the table, the regression coefficients of API, RDI, and PD are significantly positive at 1 and 5% significance levels, with estimated coefficients of 3.195, 2.319, and 0.565, respectively, while the regression coefficients of URB and OCP are significantly negative at 1 and 5% significance levels, with estimated coefficients of −1.032 and − 1.448, respectively. However, the HME regression coefficients did not meet the 10% significance test.

### 5.4 Decomposition analysis of spatial effects

It is worth noting that the SDM incorporates the influence of pertinent variables in adjacent areas; However, the coefficients of the spatial lag terms inadequately capture the true impact of these variables (131). Consequently, we calculated coefficients reflecting the direct, indirect, and total effects of API and other control variables on IRD using the estimated coefficients derived from the Time-Fixed-Effects SDM (122). The calculation results are shown in Table 3.

**Table 3:** Decomposition of direct, indirect, and total effects of the spatial durbin model with time fixed effects.

Variables	Direct-effects	Indirect-effects	Total-effects
lnAPI	3.552***	−2.848***	0.703*
	(0.441)	(0.669)	(0.418)
lnRDI	2.609***	−2.536***	0.0727
	(0.322)	(0.516)	(0.391)
LnGRE	−0.488	−0.315	−0.804
	(0.382)	(0.645)	(0.722)
LnURB	−1.432***	3.270***	1.838***
	(0.440)	(0.683)	(0.604)
LnPD	0.585***	−0.166**	0.419***
	(0.0531)	(0.0755)	(0.0481)
LnOCP	−1.405***	−0.204	−1.608***
	(0.316)	(0.443)	(0.440)
LnHME	−0.0680	−0.826**	−0.893**
	(0.246)	(0.356)	(0.362)

Standard errors in parentheses. *** *p* < 0.01, ** *p* < 0.05, * *p* < 0.1.

The direct effect analysis revealed a significant regression coefficient of 3.552 for API, indicating that air pollution contributes to increased the IRD, thereby adversely affecting public health. This is attributed to the lack of fundamental changes in China’s coal-based energy structure, leading to persistent soot-based pollution as the primary pollutant over an extended period. Meanwhile, the rising pollution from automobile exhaust has significantly heightened the risk of respiratory diseases among the population (132, 133). The results are consistent with previous studies Chen et al. (134).

Considering the control variables, the estimated coefficient of RDI for economic development is statistically significant at the 1% level, measuring 2.609. This could be related to the negative impact of air pollution on public health outweighing the positive influence of income growth among the population. Which is in line with the findings of Li et al. (135).

Regarding the living environment, the estimated coefficient of URB is statistically significant at the 1% level, with a value of −1.432. Recent studies have revealed positive trends in China’s urbanization process (136). Specifically, Chinese urbanization development policies now prioritize humanistic care and environmental protection. Moreover, Chinese urban industries are shifting from heavy pollution to green development, and urban energy consumption is becoming cleaner (137). Consequently, these developments have partially alleviated the detrimental impacts of air pollution on public health.

The estimated coefficient of PD was found to be statistically significant at the 1% level with a value of 0.585. It is because that higher PD indicates a greater likelihood of respiratory diseases among a larger population exposed to prolonged heavy air pollution (138). This observation is consistent with Ren et al. (139). The GRE demonstrated a statistically non-significant yet positive impact on public health, as evidenced by the regression coefficient of 0.488. It can be attributed to the capacity of increased the GRE to efficiently diminish, absorb, and intercept airborne toxins, including fine particulate matter and nitrogen oxides. Thus, this action significantly alleviates the detrimental impact of air pollution on public health (140). These results corroborate the conclusions drawn by Sun et al. (141).

In terms of healthcare, the estimated coefficient of the OCP was statistically significant at the 1% level, at −1.405. The possible explanation is that the increase in OCP provides favorable conditions for patients to be seen and treated, effectively mitigating air pollution-related health issues (142). The results are consistent with the findings of Yang et al. (143). In addition, HME has an insignificant positive effect on public health. The possible reason for this is that HME not only improves the physical fitness of individuals and enhances their resilience against health issues caused by air pollution but also ensures that residents have access to more adequate healthcare services, increasing the chances of addressing health issues caused by air pollution (144). The findings are similar to Zhang et al. (48).

According to the results of the indirect effect analysis, the API in the region exhibits a significant negative impact on the IRD in neighboring regions. This phenomenon likely occurs because an increase in air pollution levels in one region leads to relatively lower pollution levels in its neighboring region, thereby positively impacting public health in the latter. The RDI and HME in the region positively impact the reduction of IRD in neighboring areas. One plausible explanation for this is that the economic advancement and increased healthcare spending not only enhance the residents’ quality of life in neighboring regions but also draw nearby residents to seek medical care in the region, ensuring efficient treatment of illnesses. This finding aligns with the conclusions drawn by previous researchers Xin et al. (145). Furthermore, the rise in URB may have impeded the enhancement of public health in neighboring regions. This may stem from traditional urbanization challenges, such as intensified land development and industrial expansion, which positively correlate with IRD in adjacent areas, which consistent with Jiang et al. (146).

Furthermore, the total effect analysis revealed that increases in API, PD, and URB were significantly associated with adverse impacts on public health, whereas OCP and HME demonstrated positive effects. However, the effects of the remaining variables were deemed statistically insignificant.

### 5.5 Robustness analysis

In this study, we employ a geographic proximity matrix to assess the spatial influence of API on IRD. To mitigate potential errors in the selection process of the spatial weight matrix, we follow Feng et al. and employ a nested spatial weight matrix grounded in economic geography for robustness testing (147). The calculated results are depicted in Table 4.

**Table 4:** Robustness analysis of nested spatial weight matrix based on economic geography.

Variables	Direct-effects	Indirect-effects	Total-effects
lnAPI	1.071***	1.087	2.158***
	(0.389)	(1.330)	(1.629)
lnRDI	−0.520	−4.282	−4.802
	(0.329)	(1.521)	(1.767)
LnGRE	−1.172***	−3.609**	−4.782***
	(0.356)	(1.484)	(1.661)
LnURB	1.139**	5.945**	7.083***
	(0.470)	(2.394)	(2.713)
LnPD	0.426***	1.500***	1.926***
	(0.0390)	(0.284)	(0.308)
LnOCP	−0.0222	0.273	0.251
	(0.307)	(0.998)	(1.189)
LnHME	−0.425*	−1.963**	−2.388**
	(0.236)	(0.974)	(1.136)

Standard errors in parentheses. *** *p* < 0.01, ** *p* < 0.05, * *p* < 0.1.

From the table, it is evident that the API continues to exhibit a significant positive effect on IRD. However, the RDI and OCP show contrary results compared to the aforementioned ones. The other control variables differ only in magnitude, while the direction of spillover remains unchanged. Consequently, the robustness analysis aligns with the aforementioned analysis, indicating the stability and reliability of the paper’s findings.

### 5.6 Analysis of the spatiotemporal heterogeneity of air pollution impacts on public health

To investigate the impact of various factors on IRD across different regions and time periods, considering spatiotemporal heterogeneity, we re-evaluated the influence of API on IRD using the GTWR model implemented with Arcgis 10.8 software. Moreover, we conducted a comprehensive comparative analysis using the OLS model, the GWR model, and the GTWR model. The results are shown in Table 5.

**Table 5:** Comparison of accuracy under different models.

Model	Kernel type	Adjusted *R*^2^	AICc	Bandwidth	Observations
OLS	–	0.65	–	–	248
GWR	Gaussian	0.531	79.988	37.483	31
GTWR	–	0.98	2.557	0.113	248

From the table, we observe that the *R*^2^ value of the GTWR model (0.98) is higher than that of the OLS model (0.65) and the GWR model (0.531), indicating that the GTWR model has a better goodness of fit and stronger explanatory power. Additionally, the ARCc of the GTWR model is significantly lower than that of the GWR model, suggesting that the GTWR model is more suitable for the dataset used in this study. Therefore, we re-evaluated the influence of API on IRD using the GTWR model, implemented with ArcGIS 10.8 software.

**Figure 7:**
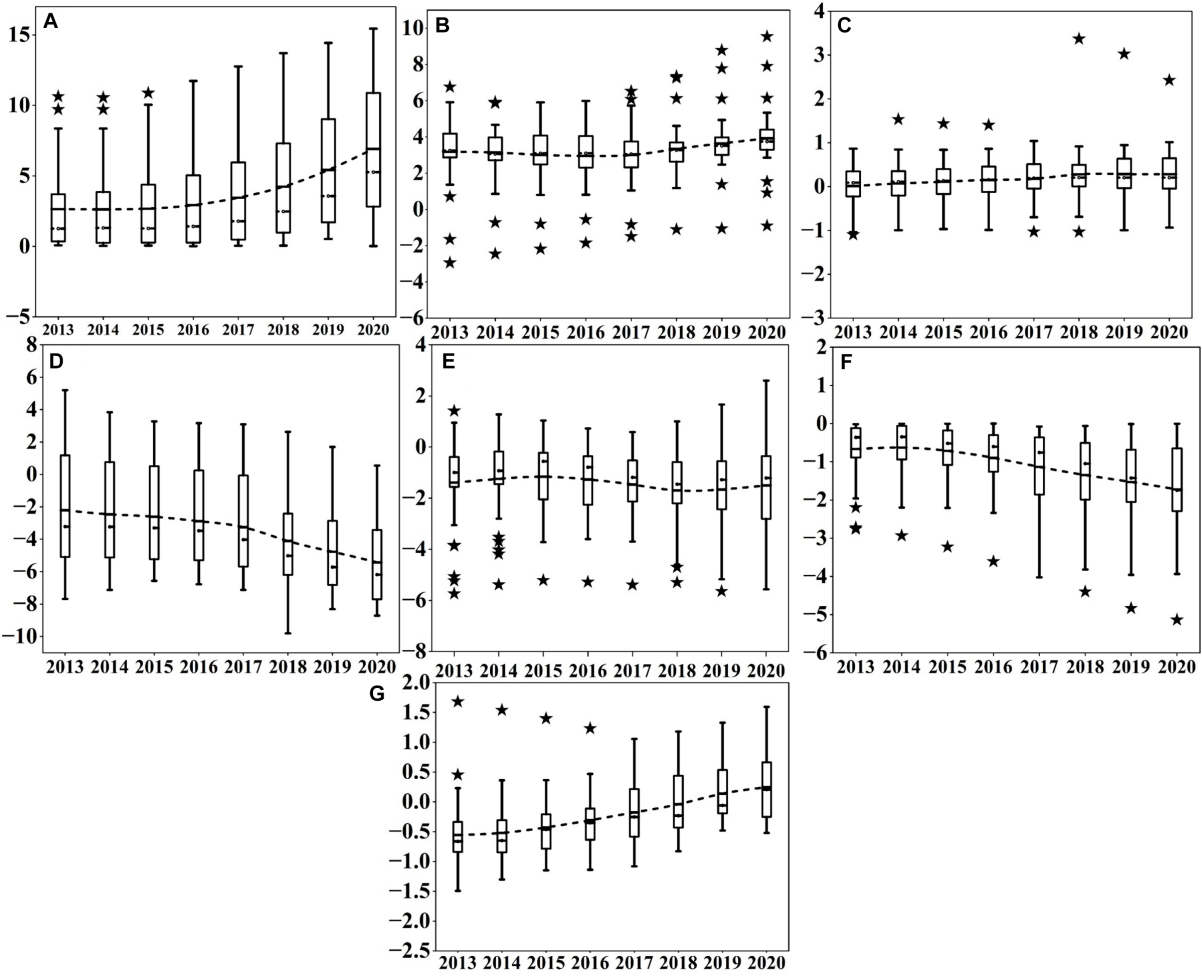
The estimated coefficients of various factors in different periods for the 31 provinces in China from 2013 to 2020. **(A)** Shows the coefficients of API; **(B)** displays the coefficients of RDI; **(C)** reflects the coefficients of PD; **(D)** represents the coefficients of URB; Figure e depicts the coefficients of GRE; **(F)** indicates the coefficients of the number of OCP; **(G)** presents the coefficients of HME.

**Figure 8:**
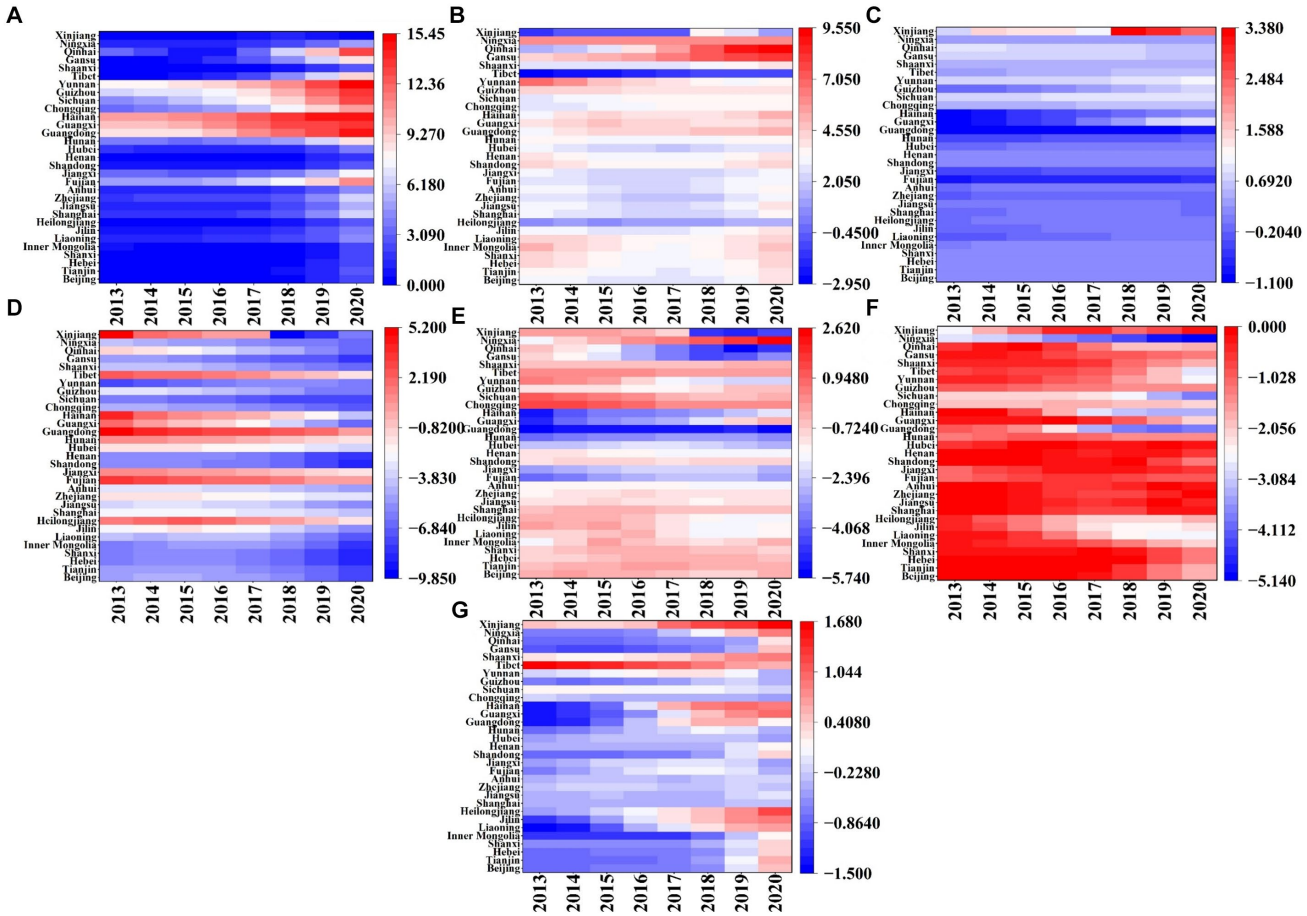
Spatial distribution of estimated coefficients of various factors in 31 provinces of China from 2013 to 2020. **(A)** Shows the coefficients of API; **(B)** displays the coefficients of RDI; **(C)** reflects the coefficients of PD; **(D)** represents the coefficients of URB; **(E)** depicts the coefficients of GRE; **(F)** indicates the coefficients of the number of OCP; **(G)** presents the coefficients of HME.

Based on the results of the GTWR model, we created Figures 7, 8 to reveal the effects of multiple factors on the spatiotemporal heterogeneity of IRD in different regions and time periods. The details are as follows.

In terms of air pollution, from the time evolution (Figure 7A), the API regression coefficients continue increasing from 2013 to 2020, indicating that the index has a positive impact on the IRD in the study area. The box becomes longer, showing that the distribution of API regression coefficients is becoming more dispersed. With regard to the spatial distribution (Figure 8A), the positive impact of API on IRD gradually decreases from 2013 to 2020 from North and Southwest China to the Eastern seaboard, with a certain gradient distribution trend. One possible explanation is that some provinces in North China, such as Hebei and Shanxi, are major coal-producing provinces, and the combustion of coal can directly generate serious air pollution. On the other hand, some of the industrial enterprises relocated from the coastal region of East China are gradually moving to North and Southwest China, leading to an increase in both energy consumption and the emission of pollutants in the region (148).

In terms of economic development, the temporal evolution of the impact degree (Figure 7B) reveals a trend where the regression coefficient of RDI initially decreases before rising again. It illustrates a predominantly positive impact of the indicator on IRD across most regions. The box diminishes in size and discreteness, demonstrating a relatively stable evolution of the RDI regression coefficient. Furthermore, numerous outliers are present in the RDI coefficients, suggesting significant variation between regions. Regarding the spatial distribution of the influence degree (Figure 8B), the extent of the positive effect of RDI on IRD from 2013 to 2020 shows an increasing trend from the East China coastal region (e.g., Zhejiang, Jiangsu, and Shanghai) to the Northwest and Southwest regions (e.g., Gansu, Ningxia, Inner Mongolia, and Guizhou). It could be explained to the increased susceptibility of residents in low-income areas such as the Northwest and Southwest regions to the impacts of air pollution. For instance, low-income groups often lack sufficient awareness about air pollution and disease prevention, and they frequently face financial constraints preventing them from covering medical and other healthcare expenses (149). Conversely, high-income groups residing in East China’s coastal areas, having satisfied their basic material needs, prioritize demanding higher environmental quality and are willing to invest more in air quality enhancements to alleviate the adverse effects of air pollution on their health (150). which is the same as the findings of Wang et al. (151).

In terms of the living environment (see Figure 7C), the coefficient of PD in regression exhibits a slight upward trend, with minimal variation within the boxplot, indicating stable dispersion. Concerning spatial distribution (Figure 8C), the overall effect of PD on IRD from 2013 to 2020 is significantly positive, with the spatial trend decreasing from the southeast coastal region to the inland northwest. Specifically, PD negatively impacts IRD most significantly in Southern and Eastern China, exemplified by Guangdong, Shandong, and Anhui, possibly due to higher population densities enabling intensive energy use, thereby reducing air pollutant emissions and favorably impacting public health (152). Conversely, IRD is mildly positively influenced by PD in northwest and southwest regions, such as the provinces and cities of Shaanxi, Gansu, and Tibet, which aligns with the findings of Chen et al. (115).

Meanwhile, from the time dimension (Figure 7D), the URB exhibits a gradual decline over time. Notable characteristics include the compact size of the box plot and decreased variability, indicating high stability. From the spatial dimension (Figure 8D), South China, represented by Fujian and Guangdong, is consistently positively correlated with URB from 2013 to 2020. This could be owed to the significant negative impact of urbanization on air quality improvement in China, thereby exacerbating public health burden (153). However, the Southwest region, including Sichuan, Guizhou, Xinjiang, and Yunnan, experienced increasing negative impacts from the URB throughout the study period. In other words, urbanization-driven population migration effectively reduces IRD (154).

Furthermore, see Figure 7E, the regression coefficients for GRE show a pattern of increase followed by decrease, indicating that this indicator has a negative impact on IRD in most regions. The boxes become more concentrated, suggesting significant variations in the distribution. Numerous outliers are present in the regression coefficient of this factor, demonstrating significant variations between the GRE coefficients and regions. From the spatial dimension (Figure 8E), the negative relationship between GRE and IRD from 2013 to 2020 exhibits a gradient distribution, ranging from high in the Middle East to low in the Northeast. Specifically, northeastern regions including Heilongjiang, Jilin, and Liaoning are weakly affected by the negative impact of GRE, while southern and central regions like Guangxi, Hunan, Guangdong, and Hubei, and eastern regions such as Shanghai, Fujian, and Zhejiang are strongly affected by the negative impact of GRE. This is related to the higher level of landscaping in South and East China compared to Northeast China (155). Landscaped green spaces can provide a variety of ecosystem services to residents, thus improving air quality and promoting public health (156), which is consistent with the findings of Yang et al. (157).

In terms of healthcare, within the temporal dimension (Figure 7F), the estimated coefficient of OCP exhibits a gradual decline, with its box plot elongating and showing increased variability, indicating significant instability in the regression coefficients. This variation may stem from significant differences in the distribution of OCP among provinces. Regarding spatial changes (Figure 8F), the negative correlation between OCP and IRD persists in East China, encompassing Guangdong, Shanghai, and Zhejiang, from 2013 to 2020. Conversely, in the southwestern and northwestern regions, including Guizhou, Sichuan, Ningxia, and Qinghai, the negative correlation between OCP and IRD gradually diminishes. One possible explanation is that the relatively more developed economic base of East China provides more adequate healthcare facilities and resources, such as modern hospitals, advanced medical equipment, and sufficient medical professionals. Which enables residents in the region to receive prompt and effective medical care when facing health problems caused by air pollution Pan et al. (158). In contrast, the Southwest and Northwest regions have a lack of local medical professionals and poor access to healthcare resources and services. Consequently, when residents in these regions are affected by air pollution, they may not receive timely medical attention, increasing the risk of illness and death (159). This finding aligns with Zhao et al. (160).

Additionally, in Figure 7G, the regression coefficients of HME exhibit a gradual upward trend, suggesting that all its effects on IRD are negative. The widening trend of its box indicates high instability in the regression coefficients. In the spatial dimension (Figure 8G), the impact of HME on IRD from 2013 to 2020 exhibits significant geographic variation. Particularly, its influence is relatively weak in economically advanced eastern regions like Jiangsu and Zhejiang. Conversely, areas with a more pronounced impact are predominantly situated in economically underdeveloped western regions, such as Heilongjiang and Gansu. This phenomenon could be attributed to the economic constraints in the western regions and the residents’ lower inclination to utilize healthcare services, consequently enabling the adverse effects of air pollution to surpass the beneficial effects of HME on public health (161). It is generally consistent with the findings of Yang et al. (149).

## 6 Conclusion

Air pollution and its effects on public health have garnered significant attention from researchers, yet there is often insufficient consideration of the spatiotemporal variations in these effects. Furthermore, the effective control of air pollution and the promotion of public health have emerged as key priorities for the Chinese government. Accordingly, based on Neidell’s theoretical framework for multifactorial health decision-making, we utilized panel data from 31 provinces and municipalities collected between 2013 and 2020. We then integrated this data with the Time-Fixed-Effects SDM and GTWR models to explore the spatiotemporal variations in the effects of air pollution on public health. This endeavor aims to furnish policymakers, urban planners, and public health authorities with actionable insights for crafting targeted interventions. The main conclusions drawn from this study are summarized below:

(1) API and IRD exhibit non-random distribution among Chinese provinces; On the contrary, they demonstrate positive spatial correlation and localized clustering. High-High clustering of API are predominantly located in Inner Mongolia, Hebei, and Liaoning, while Low-Low clustering are found in Guangdong, Guangxi, and Fujian. Similarly, High-High clustering of IRD encompass regions such as Anhui, Hunan, Hubei, and Henan, while Low-Low clustering are prevalent in Tibet, Qinghai, and other areas.
(2) The API exhibits a notable positive impact on IRD, markedly elevating IRD and thereby diminishing public health. Furthermore, the API demonstrates a more substantial adverse influence on public health compared to the traditional econometric model neglecting spatial correlation. It indicated that disregarding spatial effects may result in biased assessments of public health. Nonetheless, irrespective of spatial effects, air pollution persists as a significant determinant of public health, even after considering variables such as economic development, living environment, and healthcare. Moreover, a noteworthy spillover effect of air pollution is observed, with an indirect effect coefficient of −2.848, demonstrating that an increase in air pollution in one region can positively influence public health in neighboring provinces. Additionally, URB and OCP exhibit substantial negative impacts on IRD, while RDI and PD demonstrate significant positive effects. Conversely, GRE and HME produce insignificant negative effects on IRD.
(3) Significant spatiotemporal heterogeneity exists in the impacts of air pollution, economic development, living environment, and healthcare on IRD. Regarding temporal heterogeneity, the temporal non-stationarity of regression coefficients for API, GRE, OCP, and HME increases over time, whereas that of RDI, PD, and URB stabilizes. Regarding spatio heterogeneity, IRD in North China experiences greater influence from air pollution. Regarding economic development factor, alterations in RDI exert a more pronounced effect on IRD in northwest China. Regarding living environment factors, alterations in PD predominantly affect IRD in South and East China; Changes in URB significantly influence IRD in South and Southwest China; moreover, IRD in Northeast and East China exhibits higher susceptibility to GRE. Regarding healthcare factors, Guizhou, Sichuan, Guangdong, Shanghai, and other regions of South and Southwest China primarily experience a more significant impact from OCP on IRD. Conversely, HME primarily influences IRD in East and Northwest China.

Based on the findings of this paper, the following policy recommendations are proposed:

(i) Emphasize inter-provincial cooperation and enhance the cross-regional linkage and coordination mechanisms for air pollution prevention and control. Establish and enhance a grid-based hotspot supervision system for air pollution to bolster collaborative oversight of the air pollution control process, thereby enhancing the tangible efficacy of air pollution prevention and control efforts.
(ii) Increase investment in medical and healthcare resources and actively foster the recruitment and integration of high-quality health professionals to elevate the service standard and operational efficiency of medical and healthcare institutions. Establish a comprehensive and diverse regulatory and service framework for the medical and healthcare sector to foster a high-quality healthcare environment for patients.
(iii) Enhancing ecological and environmental protection efforts in urban and rural regions, and furthering the expansion of green spaces in both settings. Accelerating the modernization of gardening practices and green infrastructure, while implementing an effective supervision system for urban gardening and greening, to foster an enhanced ecological environment for the public.
(iv) Giving full play to the positive external effects brought about by the urbanization process, realizing the overall environmental improvement of the new urbanization, and promoting the green, healthy and sustainable development of urbanization.
(v) Owing to the spatiaotemporal heterogeneity of indicator coefficients, relevant government departments ought to consider variances in impact levels of distinct indicators while formulating strategies for air pollution prevention and control. (First) Governments in provinces and major cities like Beijing and Shanghai should not only actively foster the process of new urbanization and harness the population and industrial clustering effects it brings, but also actively promote the development of essential public services including healthcare and education, aiming to bolster public health; (Second) In provinces like Shanxi, Guangxi, Gansu, and Shaanxi, due to insufficient urban medical facilities and the uneven allocation of health resources, the government should further increase healthcare spending, actively establish a long-term inter-regional cooperation mechanism, and gradually achieve cross-regional sharing of high-quality healthcare talents, technology, knowledge, and other resources to maximize the quality of peripheral medical and healthcare services; (Third) In provinces such as Sichuan, Yunnan, Guizhou, Anhui, Zhejiang, Hunan, and Hubei, governmental institutions should establish and enhance policies for controlling air pollution, strengthen measures for environmental protection and penalties, and enhance oversight of controlling pollution from mobile sources. Additionally, governmental bodies should enhance green initiatives in urban and rural regions and rationalize the layout of green parks.

Furthermore, the limitations of this study primarily manifest in two aspects. Firstly, as data at the prefecture-level is unavailable, pertinent provincial-level data was utilized. Thus, future research could delve deeper into the effects of air pollution on public health at the prefecture level. Secondly, this paper solely concentrates on air pollution as an environmental variable. However, exploring the impacts of additional environmental pollution variables on public health will be the focus of our future investigations.

## Data availability statement

The raw data supporting the conclusions of this article will be made available by the authors, without undue reservation.

## Author contributions

YY: Writing – original draft, Writing – review & editing. QT: Supervision, Writing – review & editing. HW: Supervision, Writing – review & editing.

## Footnotes

^1^https://www.stats.gov.cn/sj/ndsj/

^2^http://www.nhc.gov.cn/wjw/index.shtml

^3^https://www.mee.gov.cn/hjzl/sthjzk/
